# Family Function Impacts Relapse Tendency in Substance Use Disorder: Mediated Through Self-Esteem and Resilience

**DOI:** 10.3389/fpsyt.2022.815118

**Published:** 2022-02-14

**Authors:** Yuwei Xia, Yu Gong, Hanbin Wang, Shen Li, Fuqiang Mao

**Affiliations:** ^1^Department of Psychiatry and Psychology, College of Basic Medical Sciences, Tianjin Medical University, Tianjin, China; ^2^Department of Mathematics, Ximou Primary School, Yantai, China

**Keywords:** substance use disorder, family function, relapse tendency, self-esteem, resilience

## Abstract

**Background:**

Substance abuse has been a public health concern, and even after detoxification treatment, the relapse rate is still high. Family function is closely related to substance dependence. However, studies on psychological mechanisms between them are rare.

**Objectives:**

We aimed to explore the mediating role of self-esteem and resilience in the pathway that family function impacts the relapse tendency among patients with substance use disorder (SUD).

**Methods:**

A total of 282 SUD patients were recruited, and standard questionnaires were administered for each patient. The relapse tendency, family function, self-esteem and resilience were assessed by the family care index questionnaire, the Connor-Davidson resilience scale, the Rosenberg self-esteem scale and the relapse tendency questionnaire. Bootstrap method was conducted for mediation analysis to test the effects of how family function affects relapse tendency mediated through self-esteem and resilience.

**Results:**

The average score of relapse tendency of the patients was 28.47 (SD = 11.89). Intermediary analysis found that self-esteem played an intermediary role in the relationship between family function and relapse tendency. Resilience plays an intermediary role in the relationship between family function and relapse tendency. Further, the path analysis showed that family function not only had a direct association with relapse tendency, but also indirectly related to relapse tendency through self-esteem and resilience.

**Conclusions:**

Self-esteem and resilience are the key factors in the relationship between family function and relapse tendency of SUD patients.

## Highlights

- The relapse tendency in substance use can be rooted in the support system from the family.
- Family function showed the influencing factors and pathways on relapse tendency of drug abusers.
- Self-esteem and resilience mediate the relapse tendency in substance use.

## Introduction

According to the “World Drug Report 2021,” more than 35 million individuals worldwide are addicted to drugs (1). In China, as of the end of 2018, there were 2.044 million drug users and 0.504 million relapsers (2). As a common and long-term recurrent mental illness (3), substance dependence poses a huge threat to human physical and mental health (4), placing a serious burden on the economy and society (5). Despite the serious adverse consequences, drug users still show obsessive drug cravings (6). Despite the best medical treatment and rehabilitation support, the relapse rate among patients with substance use disorder (SUD) remains high (7). According to reports, the relapse rate within 1 year is 40–60% (8), while it is as high as 90% under the same conditions in China (9). Relapse tendency refers to the possibility and willingness of relapse behavior, which is an important prerequisite for an individual to produce a specific behavior (10). The higher the tendency of relapse, the higher the probability of relapse behavior (11). Conventional treatment and rehabilitation mainly include medications and psychotherapy. Till now, there is no sufficient evidence to show that any drug has a significant effect in treating substance dependence and reducing the relapse rate (12). SUD patients have difficulty withdrawing and a high relapse rate, not only lying physical dependence, but also in the difficulty of getting rid of psychological dependence (13). Compared with drug therapy, psychotherapy is not only more operable, but also avoids adverse drug reactions and potential negative effects between drugs (14). Therefore, the psychological mechanisms of the relapse tendency in SUD patients warrant further study.

Family function refers to the process of providing family members with various resources to help them complete tasks (15), usually including relationship, role cognition and problem-solving (16). Family system theory (17) points out that as a dynamic whole, there are direct or indirect interactions among the various subsystems of the family, such as the husband-wife subsystem, parent-child subsystem and sibling subsystem. The family function is a common factor closely associated with substance dependence (16). Daniel et al. found that the public environment, especially within the family, would affect future drug abuse behavior. Specifically, the family function is considered to be the potential trigger of substance dependence (16). A study found that family function had a significant negative impact on substance dependence in the Iranian military (18). A longitudinal study showed that family support and encouragement play an important role in preventing relapse, and a good family environment would not only supervise them, but also enhance the guilt of patients with SUD and help reduce addictive behavior (19). The buffer model of social support argues that good social support plays a protective role in the stressful states and buffering stress (20). As the smallest unit of social groups, the family is very important to everyone. Individuals with good family support have higher levels of family function, enabling individuals to actively cope with and relieve negative emotions and stress, and reduce the tendency of relapse (21). However, the specific mechanism of the relationship between family function and relapse tendency is not clear.

Self-esteem, as a part of self-concept, is an important psychological capital. It refers to the subjective evaluation of the value of an individual as a person (22), and represents the degree of personal love and identification (23). Self-esteem is closely related to personal work, interpersonal relationships, physical and mental health (24). A study on early childhood showed that family function is essential in self-development (25). High self-esteem is positively associated with high family intimacy (26). A longitudinal study reported that family functions have a long-term and lasting impact on the development of individual self-esteem (27). People with low self-esteem often feel inferior, depressed, desperate, and even suffer from mental illness (28). In addition, individuals with low self-esteem tend to use the substance to meet their self-esteem needs, thereby controlling their emotions (29). However, positive psychological capital, such as self-esteem, is an effective protective factor against substance dependence (30). When SUD patients have a higher level of psychological capital, they maintain a more positive attitude toward life, and have a higher sense of self-efficacy. Further, they have a strong motivation for detoxification, and can resist the temptation of drugs, so their tendency to relapse will also be reduced (31). So far, there have been few studies on the role of self-esteem between family function and relapse tendency.

Resilience is the ability of individuals or groups (such as families and communities) to seek and obtain meaningful social and ecological resources, such as caregivers, peer groups or institutional support, that can protect their development in face of stress. It is an internal factor that helps individuals cope with and adapt to external pressure (32). Therefore, resilience depends on the strength of the individual and the resources provided by the favorable environment (33). It is reported that individuals with high resilience tend to face stress more flexibly and dynamically, and recover faster from negative emotions, which is an important protective factor for physical and mental health (34). Hence, more and more studies pay attention to the inherent protective factors of psychological resilience, such as self-esteem (35). High self-esteem is a protective factor of resilience, while resilience is a promoting factor of self-esteem, showing a close relationship between the two (36). Further, a cross-sectional study found that higher resilience may reduce the tendency for smoking, nicotine dependence, lifelong alcohol consumption and substance use (37). Resilience can reduce the risk of substance dependence through effective emotional regulation, tolerance of negative emotions, or actively seeking support (38). Most research on self-esteem and psychological resilience is conducted among students, not SUD patients. Studies have found that close family emotional bonds can enhance the positive psychological capital and qualities of SUD patients such as optimism, self-confidence, resilience and self-efficacy (21). Therefore, it is necessary to explore the sequential effect of self-esteem and resilience between family function and relapse tendency.

To the best of our knowledge, no studies have comprehensively explored the relationships between self-esteem, resilience, family function and relapse tendency in SUD patients from a cross-sectional perspective. In terms of the conceptualization of the intermediary mechanisms, a multiple-mediator model is more comprehensive than a single-mediator model. In our study, we aimed to explore the underlying psychological mechanisms affecting the relapse tendency in substance dependents with a chain intermediary model based on the social support buffer theory. We hypothesized that: (1) family function has a strong association with the relapse tendency among SUD patients; (2) self-esteem plays an intermediary role between family function and relapse tendency; (3) resilience mediates the effect of family function on relapse tendency; (4) the family function of strong abstinence personnel can affect their relapse tendency through the chain mediation of self-esteem and resilience.

## Materials and Methods

### Participants

Using a cross-sectional study design, an offline questionnaire survey was conducted in two drug rehabilitation centers in Tianjin, China. The group test was organized by the staff of the drug rehabilitation center with the brigade as a unit. The authenticity and confidentiality of the test were emphasized before the test, so as to reduce the impact of social approval and improve the credibility of the test results. The inclusion criteria were as follows: above 18 years old; normal and stable cognitive states; diagnosis with SUDs within the last 12 months according to Diagnostic and Statistical Manual of Mental Disorders, fifth edition (DSM-5) by experienced psychiatrists. Those with severe physical illnesses, a clear history of brain injury, other diagnoses of mental illness or under psychotherapy were excluded. The test duration was 30 min, and 282 copies of questionnaire manual were sent out. Finally, 270 valid questionnaires were returned, with an effective rate of 95.7%.

In order to explore the demographic characteristics, participants were required to fill in their age, sex, family's living status, education, type of drug use and years of drug abuse. The research protocol was approved by the Institutional Ethical Review Board of Tianjin Medical University. The informed consent was obtained from each participant.

### Family Functional Assessments

The family care index questionnaire was used to evaluate the family function, which was compiled by Smilkstein (39). There are 5 items in the questionnaire, including adaptation, partnership, growth, affection and resolve, with a score of 3 (0“rare” −2 “always”). The total score of 0–3 indicates severe family dysfunction, 4–6 is moderate and 7–10 is good. In this study, the questionnaire had a good internal consistency (Cronbach's α = 0.888).

### Self-Esteem Assessments

The Rosenberg self-esteem scale (RSES), compiled by Rosenberg (40), was conducted to assess self-esteem. There are 10 items in the scale and 4 points are used to score each item (1 “very inconsistent” −4 “very consistent”). The higher total score means the higher levels of self-esteem. In our current study, the questionnaire had a good internal consistency (Cronbach's α = 0.832).

### Psychological Resilience Assessments

The Connor-Davidson resilience scale (CD-RISC) was performed to assess the psychological resilience. The Chinese version of the Connor-Davidson psychological resilience scale (41) was used, with a total of 25 items, including tenacity, self-improvement and optimism. Each item has a score with 5-point (0“never” -4 “always the same”). The higher the total score indicates the higher levels of the psychological resilience. Also, the internal consistency of this questionnaire is good (Cronbach's α = 0.964).

### Relapse Tendency Assessments

The psychological questionnaire of relapse tendency of SUD patients was used, compiled by Geng Wenxiu (42). This scale consists of 18 items, including self-assessment of confidence in detoxification, current drug influence, objective environment, degree of physical and mental damage, and support system, with a score of 6-level (0“almost impossible” −5 “very easy”). The higher the total score, the higher the tendency of relapse. In this study, there was a good internal consistency of this questionnaire (Cronbach's α = 0.856).

### Statistical Analysis

Firstly, each variable was checked for normality using Kolmogorov Smirnov one-sample test. Descriptive statistics were performed to report the scores of demographic variables and family function, self-esteem, resilience and relapse tendency. Independent sample *t*-tests and one-way analysis of variance (ANOVA) were used to investigate whether there were differences in relapse tendency among respondents with different demographic characteristics. To explore the relationship among the four variables of family function, relapse tendency, self-esteem and resilience, we used Pearson correlation analysis. Further, the intermediary analysis was carried out by using the bootstrap method (43) in the PROCESS program. Prior to analyses, all variables have been standardized. In the intermediary analysis, relapse tendency was the outcome variable, family function was the independent variable, self-esteem and resilience were entered as mediating variable, including age, gender, education, drug use, years of abuse use and living status as covariates. Moreover, bootstrapping (5,000 bootstrap samples) with 95% confidence intervals (CIs) was conducted to examine the significance of the mediation and moderation effects; 95% CIs that do not contain zero indicate that the effects are significant. All above statistical analyses were carried out using SPSS25.0 software.

## Results

### Sociodemographic Characteristics

In the 270 valid samples, there were 125 males and 145 females, aged from 21 to 60 years old (36.20 ± 8.55). 64.1% came from cities and 35.9% came from rural areas. Among them, 18.9% were educated in primary school or below, 44.8% have a junior middle school degree, 21.1% have a high school degree and 15.2% have university degree or above (Table 1). The relapse tendency of female patients was higher than that of male patients (33.18 ± 9.45 vs. 29.24 ± 16.63, *t* = −2.43, *p* = 0.016); and patients in urban showed higher relapse tendency than rural patients (33.33 ± 13.49 vs. 25.25 ± 11.37, *t* = 4.41, *p* < 0.001) (Supplementary Table 1). Age, education, type of drug use and years of drug abuse did not show significant differences in relapse tendency.

**Table 1:** Sociodemographic characteristics (*N* = 270).

**Characteristics**		***n***	**%**
Age (years)	Min–max	21–60	
	Mean (SD)	36.20 (8.55)	
Gender	Male	125	46.30
	Female	145	53.70
Area of family residence	Urban	173	64.07
	Rural	97	35.93
Education	Primary school or lower	51	18.89
	Junior middle school	121	44.81
	High school	57	21.11
	University or above	41	15.19
Type of drug use	Heroin	48	17.8
	Methamphetamine	220	81.5
	Other drugs	2	0.7
Years of drug abuse	One year	23	8.5
	Two years	18	6.7
	Three years	38	14.1
	Four years	26	9.6
	Five years and more	165	61.1

### The Correlations Between Family Function, Self-Esteem, Resilience, and Relapse Tendency

Descriptive analysis showed that the scores of each variable of drug abusers were as follows: family function score (6.06 ± 1.32), self-esteem score (19.50 ± 6.11), resilience score (46.39 ± 18.09), relapse tendency score (28.47 ± 11.89).

As shown in Table 2, family function was positively correlated with self-esteem (*r* = 0.791, *p* < 0.001) and resilience (*r* = 0.847, *p* < 0.001), and negatively correlated with relapse tendency (*r* = −0.907, *p* < 0.001). There was a significant positive correlation between self-esteem and resilience (*r* = 0.817, *p* < 0.001), and a significant negative correlation between self-esteem and relapse tendency (*r* = −0.810, *p* < 0.001). In addition, resilience was negatively correlated with relapse tendency (*r* = −0.860, *p* < 0.001).

**Table 2:** Descriptive statistics and Pearson correlations between all variables (*N* = 270).

	**Scores**	**1**	**2**	**3**	**4**
1 family function	6.06 ± 1.32	1			
2 self-esteem	19.50 ± 6.11	0.791^***^	1		
3 resilience	46.39 ± 18.09	0.847^***^	0.817^***^	1	
4 relapse tendency	28.47 ± 11.89	−0.907^***^	−0.810^***^	−0.860^***^	1

*** *p < 0.001*.

### The Mediating Effects of Resilience and Self-Esteem in the Relationship Between Family Function and Relapse Tendency

First of all, the effect of family function on relapse tendency of SUD patients was analyzed. As shown in Table 3, family function had a significantly negative association with relapse tendency (β = −7.889, *p* < 0.001). Then, the intermediary role of self-esteem and resilience in the influence of family function on relapse tendency was analyzed. In this step, family function had significant negative and direct effect on relapse tendency (β = −5.144, *p* < 0.001), self-esteem had significant negative effect on relapse tendency (β = −0.283, *p* < 0.01), and resilience had significant negative effect on relapse tendency (β = −0.162, *p* < 0.001). In addition, family function had a significant positive effect on self-esteem (β = 3.295, *p* < 0.001) and resilience (β = 6.175, *p* < 0.001), and self-esteem had a significant positive effect on resilience (β = 1.520, *p* < 0.001). From the results above, family function was directly related to relapse tendency, and it can also affect relapse tendency through the mediation of self-esteem, resilience and chain mediation of both self-esteem and resilience.

**Table 3:** The results from mediation analysis using a bootstrapping method for relapse tendency.

**Regression equation**		**Integral fitting index**			**Significance of** **regression coefficient**		**Collinearity** **Statistics**	
**Outcome**	**Predictors**	**R**	**R^2^**	**F**	**β**	***t***	***TOL***	***VIF***
Relapse tendency	Family function	0.932	0.869	247.735^***^	−7.889	−39.231^***^	0.943	1.060
Self-esteem	Family function	0.834	0.696	85.503^***^	3.295	22.702^***^	0.943	1.060
Resilience	Family function	0.896	0.803	133.188^***^	6.175	9.668^***^	0.318	3.146
	Self-esteem				1.520	9.630^***^	0.304	3.284
Relapse tendency	Family function	0.948	0.900	258.794^***^	−5.144	−14.514^***^	0.234	4.272
	Self-esteem				−0.283	−3.233^**^	0.225	4.451
	Resilience				−0.162	−5.499^***^	0.297	5.082

** *p < 0.01*,

*** *p < 0.001*.

The significance of the intermediary effect was tested by Bootstrap analysis, as shown in Table 4. The results showed that the direct effect of family function on relapse tendency, the mediating effect of self-esteem, the mediating effect of resilience, the chain mediating effect of self-esteem and resilience were significant (the confidence interval did not include 0). The direct effect accounts for 65.20% of the total effect, while the total indirect effect accounts for 34.80%. At the same time, the intermediary effect of self-esteem accounts for 11.81%, the intermediary effect of resilience accounts for 12.69%, of which the chain intermediary effect of self-esteem and resilience accounts for 10.30%.

**Table 4:** Indirect effect of family function on relapse tendency via self-esteem and resilience.

**Path**	**Coefficient**	**Relative** **effect (%)**	**95% confidence** **interval**	
			**Boot** **LLCI**	**Boot** **ULCI**
Total effect	−7.889		−8.285	−7.493
Direct effect	−5.144	65.20	−5.842	−4.446
Total indirect effect	−2.745	34.80	−3.425	−2.120
Family function → self-esteem → relapse tendency	−0.932	11.81	−1.607	−0.322
Family function → resilience → relapse tendency	−1.001	12.69	−1.481	−0.606
Family function → self-esteem → resilience → relapse tendency	−0.812	10.30	−1.143	−0.493

*LLCI, Lower limit of confidence interval; ULCI, Upper limit of confidence interval*.

**Figure 1:**
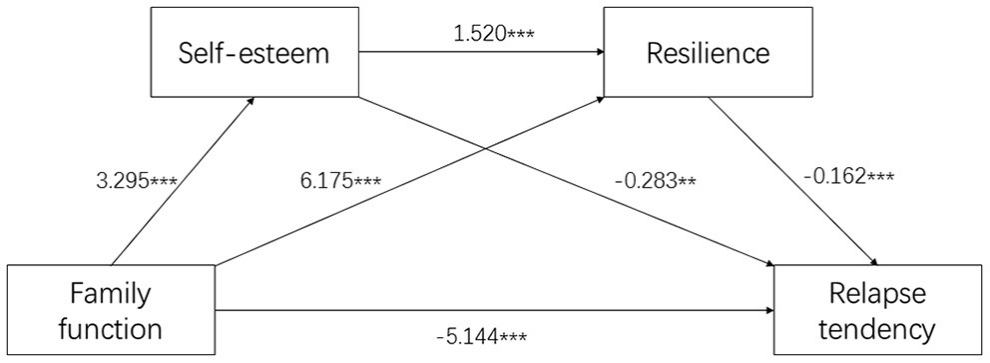
Path analysis for Family Function-Self-Esteem-Resilience-Relapse Tendency Model. ^**^p < 0.01; ^***^p < 0.001; Values are shown as standardized coefficients.

Based on the above results, the path analysis of the relationship between the four variables is carried out. Figure 1 shows that the model fits well with the whole sample data.

## Discussion

Our study provides novel evidence that resilience and self-esteem are important protective factors between family functioning and relapse tendency. We found that family function not only directly affects the relapse tendency, but also indirectly affects it through the mediation of self-esteem and resilience. The mediation is achieved through the following three paths: (1) the independent intermediary role of self-esteem; (2) the independent intermediary function of resilience; (3) the chain mediation of self-esteem and resilience.

Intermediary effect analysis showed that family function could positively associate with the relapse tendency in SUD patients, which was consistent with the previous study (31). Their family functions are poor, which leads to deficits in self-control (44). The degree of family care, intimacy and support are poor, making it difficult for family members to learn effective social communication and positive coping styles, thereby obtaining less social support and exhibiting a degree of aggression (45). Additionally, they lack the psychological resources and social support to resist drug cravings when they face negative life events or difficulties (46, 47). Also, they lack positive ways to solve the problem. It is easy to temporarily evade or reduce negative emotional experiences such as anxiety, helplessness and depression by retaking drugs (48). In addition, it is believed that there is a bidirectional relationship between family function and drug abuse. When there is a SUD patient in the family, the family relationships will be materially and negatively affected (49), such as blame, anger and even alienation. The increase of family conflicts and family dysfunction (50), in turn, prompted these patients to relieve stress and negative emotions through relapse, creating a vicious circle.

Then, self-esteem and resilience were entered as intermediary variables to explore the psychological mechanisms of family function and relapse tendency. In our results, the family function could influence the relapse tendency of SUD patients through the intermediary role of self-esteem, with the effect of 13.23%. Such patients have poor family functions, and their families cannot serve the members' self-development (51). They feel that they have not been supported by their families, with a low level of self-esteem, as well as negative self-cognition and self-evaluation. Under the negative emotional state, they usually lack confidence in detoxification, which increases the tendency of relapse.

The family function could affect the relapse tendency through the intermediary role of resilience, with an effect of 14.66%. This finding is also consistent with the previous study (52). Kaplan et al. believed that family is one of the important protective factors for resilience (53), also supported by some other studies (54, 55). An intervention study on SUD patients showed that interventions in their resilience could effectively reduce the recurrence rate (56). The family functions of SUD patients are poor (57). When individuals encounter setbacks or adversity, it is difficult to obtain support from their families, and thus they are unable to possess resilience. The lack of resilience usually means that it is more difficult for individuals to solve the problems in face of stressful situations. At the same time, studies have confirmed that individuals are at a greatly increased risk of substance dependence in stressful situations (58). Thus, the family function may influence the relapse tendency through the intermediary role of self-esteem and resilience in SUD patients.

Furthermore, we also found that family function can influence the tendency of relapse through a chain mediation of “self-esteem-resilience.” Specifically, the family functions of SUD patients are dysfunctional, which prevents them from receiving support. As a result, they have a low evaluation of themselves, leading to a state of low self-esteem. At the same time, individuals with low levels of self-esteem cannot always face problems correctly and solve them actively, making them unable to recover quickly when they encounter difficulties. This means that their resilience is very poor. Also, people with low resilience tend to take drugs to get rid of problems and difficulties temporarily.

## Limitations

Our study elucidated the psychological mechanisms of family function on relapse tendency in SUD patients, and clarified the chain intermediary role of self-esteem and resilience. At the same time, it also emphasizes that the roots of the relapse tendency of individuals could be traced back to the various subsystems of the family. As factors closely related to the relapse tendency, self-esteem and resilience deserve the attention of the researchers and the rehabilitators.

There were several limitations to the current study. First, the psychological indicators of SUD patients were evaluated through self-reports, and were prone to memory bias. Second, this study is a cross-sectional design, and the causal conclusion cannot be drawn. Third, due to the particularity of the survey population, the small sample size of this study limited our ability to address our hypotheses. Fourth, some other information was not collected, such as addiction severity, drug use trajectories, income, and details of legal drug use, which were helpful to render the data interpretable. Finally, all the recruited SUD patients were from Tianjin, so it is unknown whether the conclusion can be extended to and settings. Future studies using a high-quality longitudinal study in an independent large sample are required and helpful to illuminate these connections among family function and relapse tendency.

## Conclusion

To sum up, this study has shown that the relapse tendency of SUD patients is directly related to their family function, and indirectly through the mediation of self-esteem and resilience. Our findings suggest that interventions at the family level can be attempted to reduce the likelihood of relapse tendency during the detoxification treatment in SUD patients.

## Data Availability Statement

The raw data supporting the conclusions of this article will be made available by the authors, without undue reservation.

## Ethics Statement

The studies involving human participants were reviewed and approved by the Institutional Ethical Review Board of Tianjin Medical University. The patients/participants provided their written informed consent to participate in this study.

## Author Contributions

FM and SL were responsible for study supervision. YX was responsible for study concept and design and drafting of the manuscript. YG was involved in evolving the ideas and editing the manuscript. HW collected the data. All authors have contributed to and have approved the final manuscript.

## Funding

This study was supported by the Preponderant Education and Treatment Project of Tianjin Drug Detoxification Bureau (Grant No. 2401/1RW017).

## Conflict of Interest

The authors declare that the research was conducted in the absence of any commercial or financial relationships that could be construed as a potential conflict of interest.

## Publisher's Note

All claims expressed in this article are solely those of the authors and do not necessarily represent those of their affiliated organizations, or those of the publisher, the editors and the reviewers. Any product that may be evaluated in this article, or claim that may be made by its manufacturer, is not guaranteed or endorsed by the publisher.
