# Supplementary material for: Family Function Impacts Relapse Tendency in Substance Use Disorder: Mediated Through Self-Esteem and Resilience
Source: Front Psychiatry. 2022 Feb 14;13:815118. doi: 10.3389/fpsyt.2022.815118 (PMC8882822; doi:10.3389/fpsyt.2022.815118)
Supplement: Supplementary file 1 [file Table_1.docx]

**Family function impacts relapse tendency in substance use disorder: mediated through self-esteem and resilience**

**Yuwei Xia^1†^, Yu Gong^1†^, Hanbin Wang^2^, Shen Li^1*^, Fuqiang Mao^1*^**

^1^College of Basic Medical Sciences, Department of Psychiatry and Psychology, Tianjin Medical University, Tianjin, China

^2^Ximou Primary School, Yantai, China

^†^ These authors contributed equally to this article.

*** Correspondence:**Fuqiang Mao; Department of Psychiatry and Psychology, College of Basic Medical Sciences, Tianjin Medical University, 22 Qixiangtai Road, Heping District, Tianjin, 300070, China. Tel: 86-22-83336853; Email：[maofq@tmu.edu.cn](mailto:maofq@tmu.edu.cn)

Shen Li; Department of Psychiatry and Psychology, College of Basic Medical Sciences, Tianjin Medical University, 22 Qixiangtai Road, Heping District, Tianjin, 300070, China. Tel: 86-22-83336853; Email：[lishen@tmu.edu.cn](mailto:lishen@tmu.edu.cn)

**Words in text:** 3505 excluding abstract, references, tables and figures

**Words in abstract:** 241

**Reference:** 59

**Tables:** 4

**Figure:** 1

**Supplementary materials:** 1

***Supplemental Materials***

**Supplemental Table 1 |** Gender and area of family residence differ in relapse tendency (N= 270).

|  |  | ***t*** | ***p*** |
| --- | --- | --- | --- |
| Gender | Male | -2.43 | 0.016 |
|  | Female |  |  |
| Area of family residence | Urban | 4.41 | <0.001 |
|  | Rural |  |  |
